# Association of serum 25-hydroxyvitamin D (25(OH)D) levels with the gut microbiota and metabolites in postmenopausal women in China

**DOI:** 10.1186/s12934-022-01858-6

**Published:** 2022-07-11

**Authors:** Jinhua Gong, Lina He, Qinyuan Zou, Yangyang Zhao, Bangzhou Zhang, Rongmu Xia, Baolong Chen, Man Cao, Wenxiu Gong, Lin Lin, Xiujuan Lin, Guowei Wang, Muyun Guo, Jianquan He, Chuanxing Xiao, Jian Chen

**Affiliations:** 1 Xiamen Institute of Union Respiratory Health, Xiamen, China; 2 Key Laboratory for Research on Active Ingredients in Natural Medicine of Jiangxi Province, Yichun University, Yichun, China; 3 School of Medicine, Xiamen University, Xiamen, China; 4 School of Pharmacy, Fujian University of Traditional Chinese Medicine, Fuzhou, China; 5 Department of Gastroenterology, The Second Affiliated Hospital of Fujian University of Traditional Chinese Medicine, Fuzhou, China; 6 Xiamen Treatgut Biotechnology Co., Ltd., Xiamen, China; 7 Institute of Basic Theories of Chinese Medicine, China Academy of Chinese Medical Sciences, Beijing, China; 8 Pulmonary and Critical Care Medicine, Anyang District Hospital, Anyang, China; 9 College of Rehabilitation Medicine, Fujian University of Traditional Chinese Medicine, Fuzhou, China; 10 Department of Rehabilitation, Zhongshan Hospital of Xiamen University, School of Medicine, Xiamen University, Xiamen, China; 11 School of Basic Medical Science, Central South University, Changsha, China

**Keywords:** 25-hydroxyvitamin D, Gut microbiota, Gut metabolite, Postmenopausal women, 16S rRNA gene sequencing, LC–MS metabolomics

## Abstract

**Background:**

Vitamin D insufficiency or deficiency is associated with an altered microbiota in older men. However, the relationship between the gut microbiota and 25-hydroxyvitamin D (25(OH)D) levels remains unknown in postmenopausal women. In this study, fecal microbiota profiles for 88 postmenopausal women in the high 25(OH)D (HVD) group (n = 44) and the low 25(OH)D (LVD) group (n = 44) were determined. An integrated 16S rRNA gene sequencing and liquid chromatography–mass spectrometry (LC–MS)-based metabolomics approach was applied to explore the association of serum 25(OH)D levels with the gut microbiota and fecal metabolic phenotype. Adjustments were made using several statistical models for potential confounding variables identified from the literature.

**Results:**

The results demonstrated that the community diversity estimated by the Observe, Chao1 and ACE indexes was significantly lower in the LVD group than in the HVD group. Additionally, two kinds of characteristic differences in the microflora were analyzed in the HVD group, and ten kinds of characteristic differences in the microflora were analyzed in the LVD group. We observed that some bacteria belonging to the genera *Bifidobacterium*, *Bacillus*, *F0332* and *Gemella*, were enriched in the LVD group, as were other genera, including *Lachnoclostridium*, *UC5*_*1*_*2E3, Ruminococcus_gnavus_group* and un_f_Lachnospiraceae. *Christensenellaceae*, *Eggerthellaceae* and *Cloacibacillus* were enriched in the HVD group. The L-pyroglutamic acid, inosine, and L-homocysteic acid levels were higher in the HVD group and were negatively correlated with the 1,2-benzenedicarboxylic acid and cholic acid metabolic levels.

**Conclusions:**

These observations provide a better understanding of the relationships between serum 25(OH)D levels and the fecal microbiota and metabolites in postmenopausal women.

**Supplementary Information:**

The online version contains supplementary material available at 10.1186/s12934-022-01858-6.

## Introduction

Vitamin D (VD) insufficiency or deficiency is a frequent comorbidity in Chinese women with postmenopausal osteoporosis (PMO) [1]. Osteoporosis is a chronic, systemic skeletal disease with increased susceptibility to brittle bones and fractures; 200 million people worldwide are affected, and 50% of postmenopausal women suffer an osteoporosis-related fracture during their lifetime [2, 3]. VD maintains calcium/phosphate homeostasis and is widely used to prevent and treat osteoporosis, and its concentration in the body, which is partly obtained from food sources, depends largely on 7-dehydrocholesterol (vitamin D_3_) being converted by ultraviolet irradiation in the skin [4]. In addition, VD deficiency is associated with nonskeletal diseases such as cardiovascular disease, cancer and diabetes [5–7]. The major sources of VD are sun exposure, diet, and supplements [8]. The problem, however, is that the ability to synthesize VD in the skin becomes decreasing with advancing age. Therefore, with increasing age, VD adequacy relies increasingly on dietary intake [9].

VD is metabolized by first undergoing hydroxylation by the enzyme 25-hydroxylase to form 25-hydroxyvitamin D_3_. In the kidneys, the enzyme 1α-hydroxylase (CYP27B1) forms an active form of VD, namely, 1,25-dihydroxyvitamin D_3_ (1,25(OH)_2_D_3_) [10]. Eventually, 1,25(OH)_2_D_3_ is metabolized by the renal CYP24A1 enzyme in the kidney [11]. Clinically, serum 25-hydroxyvitamin D (25(OH)D) levels ≥ 20 ng/ml are considered adequate, while serum 25(OH)D levels < 20 ng/mL are defined as VD deficiency [12]. VD deficiency alters the microbiome and integrity of the gut epithelial barrier. Changes in serum levels of the 25(OH)D post-supplementation vary widely among individuals, with around 25% of people demonstrating no response [13–16]. The responder and non-responder groups differed with respect to changes in gut microbial composition post-supplementation [17].The trillions of microorganisms in the gastrointestinal tract are important factors in maintaining human health and are involved in inflammatory, metabolic, cardiovascular, autoimmune, neurological and psychiatric diseases [18]. The vitamin D receptor (VDR) is highly expressed on the intestinal epithelium and binds to 25(OH)D, which is a key player in intestinal homeostasis by regulating mucosal immunity [19].

VD also changes the microbiome, and manipulation of bacterial abundance or composition impacts disease manifestation [20]. Oral vitamin D_3_ supplementation influences the human gut microbiome of the upper gastrointestinal tract and is associated with reduced opportunistic pathogens and increased bacterial richness [21]. VD and the VDR adjust the innate immune response to the microbiome by controlling microbiota dysbiosis and maintaining tolerance in the gut [22]. Therefore, VD status is connected with the composition and function of the intestinal microflora.

To date, several studies have reported that the intestinal microbiota plays a key role in the pathogenesis of PMO and represents a new target for treating PMO [23]. Nonetheless, the evidence of an association between 25(OH)D levels and the gut microbiota in postmenopausal women remains inadequate. Our aim in the present study was to investigate whether intestinal microbiota features are associated with 25(OH)D levels using 16S rRNA gene sequencing and liquid chromatography–mass spectrometry (LC/MS)-based metabolomics to explore an alternative strategy to enhance calcium absorption as a strategy for osteoporosis prevention. It is of significant importance for preventing osteoporosis among the elderly or among people living in areas with little sunshine.

## Materials and methods

### Human participants

Ethical approval was granted by the Ethics Committee of Zhongshan Hospital of Xiamen University (No. 201808) before recruitment. Postmenopausal women were recruited from Xiamen, Fujian Province, China, between December 1, 2018, and February 1, 2019. Postmenopausal status was defined as at least one year since the last menstruation. Written informed consent was obtained from all the participants. We adopted the following exclusion criteria before fecal sample collection: (1) use of antibiotics or hormones within 3 months; (2) consumption of pro/pre/synbiotic products within two weeks; (3) use of medications (e.g., calcium, VD, calcitriol, alpha calcitriol, estrogen, glucocorticoids, diphosphonate, denosumab or teriparatide); and (4) presence of bone disease, hyperthyroidism, hypothyroidism, gastrointestinal disease, cancer, kidney disease, or mental illness or evidence of recent infections.

### Determination of clinical parameters

The basic information of all the subjects, such as age, height and weight, was collected, and the body mass index (BMI) was calculated according to the data of the latter two items. Blood samples were collected from all the subjects in a fasting state and at similar time points in the morning. The levels of serum 25(OH)D, estradiol (E2), osteocalcin (OC), C-terminal telopeptide of type I collagen (CTX-I), procollagen type 1 n-terminal propeptide (P1NP), and parathyroid hormone (PTH) were measured with an automated Roche Osteoporosis Int electrochemiluminescence system (Roche Diagnostics GmbH, Germany). The inter- and intra-assay coefficients of variation (CVs) were 8.0% and 5.6% for 25(OH)D, 2.9% and 2.3% for E2, 4.0% and 2.9% for OC, 3.5% and 2.5% for CTX-1, 2.8% and 2.3% for P1NP, and 2.9% and 1.7% for PTH, respectively. The bone mineral densities (BMDs) of the lumbar spine (LS) (L1-4) and total hip joint (femoral neck (FN), trochanter, and intertrochanteric region) were measured with a daily calibrated Hologic 4500 A dual-energy X-ray absorptiometry scanner (Lunar Expert 1313, Lunar Corp, USA).

### Sequencing and bioinformatics

Fecal samples were collected in sterile plastic cups, frozen, and stored at − 80 °C within 1 h until further processing [24]. Fecal microbial DNA was extracted using a QIAamp DNA Stool Mini Kit (Qiagen, Hilden, Germany). PCR amplification was carried out using an ABI 2720 Thermal Cycler (Thermo Fisher Scientific, USA). We used Multiskan™ GO spectrophotometry (Thermo Fisher Scientific, USA) to quantify bacterial genomic DNA as the template for amplification of the V3-V4 hypervariable region of the 16S rRNA gene in three replicate reactions with forward (Illumina adapter sequence 5′-CCTACGGGNBGCASCAG-3′) and reverse (Illumina adapter sequence 5′-GGACTACNVGGGTWTCTAAT-3′) primers. Replicate PCR products were pooled and purified with Agencourt AMPure XP magnetic beads (Beckman Coulter, USA). A TopTaq DNA Polymerase kit (Transgen, China) was used. The purity and concentration of sample DNA were assessed using a NanoDrop 2000 Spectrophotometer (Thermo Fisher Scientific, USA). Paired-end sequencing was performed by Treatgut Biotechnology Co., Ltd. with a HiSeq 2500 (Illumina, San Diego, CA, USA) with PE 250 bp reagents.

After sequencing, raw paired-end reads were assembled using FLASH [25] with the default parameters. Primers were removed using cutadapt, and clean tags were obtained by removing the lower reads using cutadapt [26]. To assign de novo operational taxonomic units (OTUs), we removed chimeric sequences and clustered sequences with 97% similarity and used Usearch (V10.0.240) [27] for the study. The representative sequences of OTUs were aligned to the SILVA132 database for taxonomic classification by RDP Classifier [28] and aggregated to various taxonomic levels.

### Fecal metabolite extraction

At least fifty milligrams of sample was placed in an EP tube, and then 1000 μL of extraction liquid containing an internal target (V methanol:V acetonitrile:V water = 2:2:1, which was kept at − 20 °C before extraction) was added. The samples were homogenized in a bead mill for 4 min at 45 Hz and ultrasonicated for 5 min (incubated in ice water). After homogenization 3 times, the samples were incubated for 1 h at − 20 °C to precipitate proteins. The samples were centrifuged at 12,000 rpm for 15 min at 4 °C. The supernatant (750 μL) was transferred to fresh EP tubes, and the extracts were dried in a vacuum concentrator without heating. Then, 100 μL of extraction liquid (V acetonitrile:V water = 1:1) was added for reconstitution. The samples were vortexed for 30 s, sonicated for 10 min (4 °C water bath), and centrifuged for 15 min at 12,000 rpm and 4 °C. The supernatant (60 μL) was transferred to a fresh 2 mL LC/MS glass vial, and 10 μL was collected from each sample and pooled as quality control (QC) samples. Sixty microliters of supernatant was used for ultra-high-performance liquid chromatography combined with quadrupole time-of-flight mass spectrometry (UHPLC-QTOF-MS) analysis.

### LC–MS/MS analysis and annotation

LC–MS/MS analyses were performed using a UHPLC system (1290, Agilent Technologies) with a UPLC BEH Amide column (1.7 μm 2.1 × 100 mm, Waters) coupled to a TripleTOF 6600 (Q-TOF, AB Sciex) & QTOF 6550 (Agilent). The mobile phase consisted of 25 mM NH4OAc and 25 mM NH4OH in water (pH = 9.75) (A) and acetonitrile (B), which was applied in an elution gradient as follows: 0 min, 95% B; 7 min, 65% B; 9 min, 40% B; 9.1 min, 95% B; and 12 min, 95% B, which was delivered at 0.5 mL/min. The injection volume was 2 μL. A TripleTOF mass spectrometer was used due to its ability to acquire MS/MS spectra on an information-dependent basis (IDA) during an LC/MS experiment. In this mode, the acquisition software (Analyst TF 1.7, AB Sciex) continuously evaluates the full-scan survey MS data as it collects and triggers the acquisition of MS/MS spectra depending on preselected criteria. In each cycle, 12 precursor ions with intensities greater than 100 were chosen for fragmentation at a collision energy of 30 V (15 MS/MS events with a product ion accumulation time of 50 ms each). The ESI source conditions were set as follows: ion source gas 1 at 60 psi, ion source gas 2 at 60 psi, curtain gas at 35 psi, source temperature at 650 °C, and ion spray voltage floating at 5000 V or − 4000 V in positive or negative modes, respectively.

MS raw data files were converted to mzXML format using ProteoWizard [29] and processed with the R package XCMS (version 3.2). The preprocessing results generated a data matrix that consisted of the retention time (RT), mass-to-charge ratio (m/z) values, and peak intensity. The R package CAMERA was used for peak annotation after XCMS data processing [30].

### Statistical analyses and visualization

The rarefaction curves constructed from the sequenced data has been basically stable, indicating that the sequenced data has benn basically stable at this sequencing depth (Additional file 1: Fig. S1). The alpha diversity indexes, bacterial richness (observed OTUs), Shannon, Simpson, ACE, Chao1 index and evenness (J) were calculated based on OTU tables of the study. Significance tests between the HVD and LVD groups were conducted with the Wilcoxon test method. Differences in community structure across samples (beta diversity) were visualized by principal coordinates analysis (PCoA) plots based on Bray–Curtis distance. Significance tests were determined using permutational multivariate analysis of variance (PERMANOVA) with 999 permutations in vegan [31]. Linear discriminant analysis effect size [32] was performed to identify taxa with differential abundance between the HVD and LVD groups. We further explored the correlation between different genera and fecal metabolites by Spearman’s rank test. To evaluate functional differences in the gut microbiomes of the HVD versus LVD groups, we performed PICRUSt [33] to calculate the microbial abundances, assign metabolic pathways to the gut microbiomes using KEGG and COG, and then test the differences between the two groups. All statistical and correlational analyses were conducted in R (v3.6.0) [34]. Figures were plotted mainly using ggplot2 (v3.0.0) [35].

## Results

### Characteristics and clinical and biochemical indexes of the participants

**Fig. 1:**
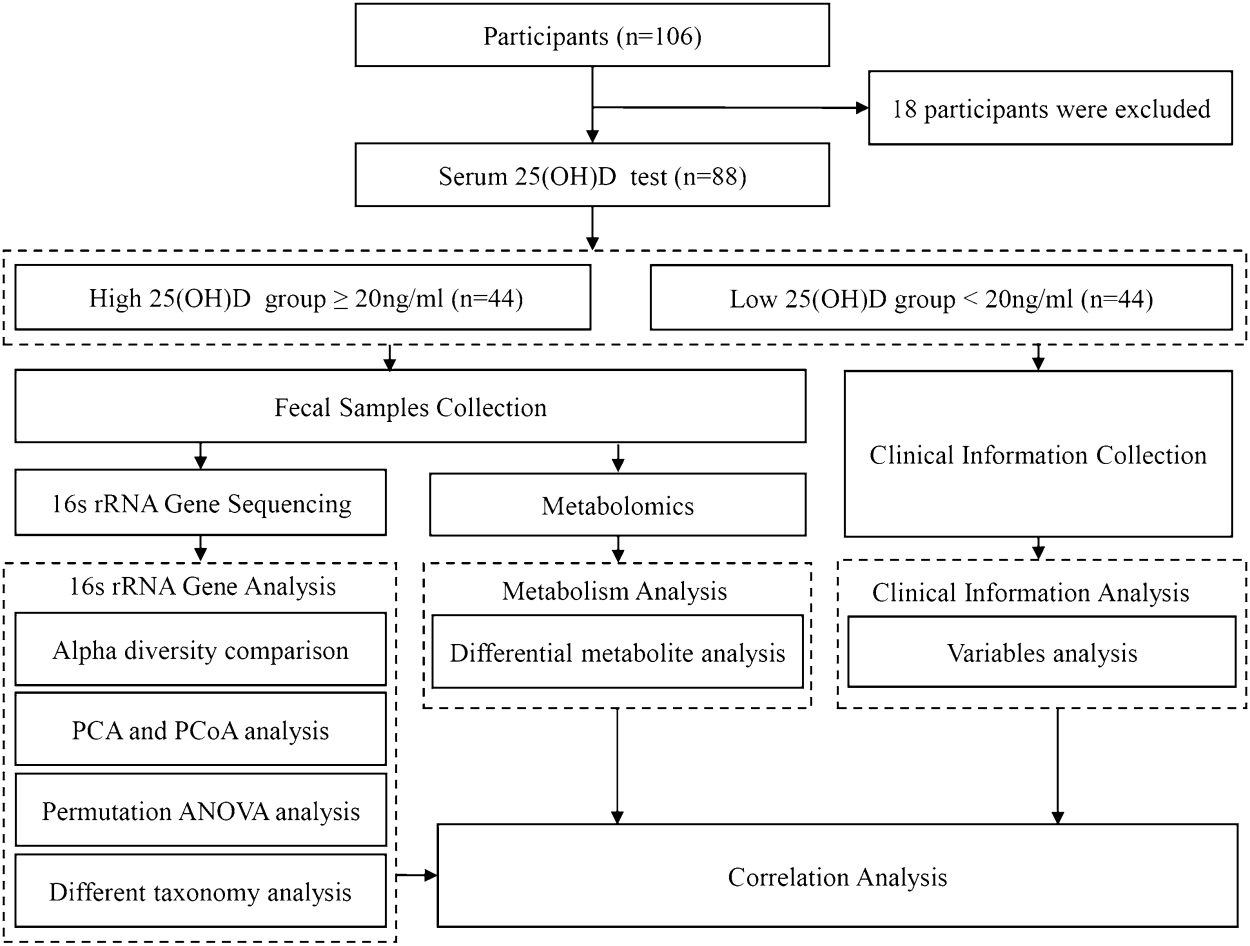
Flow diagram of this study

According to the inclusion and exclusion criteria, 18 patients were excluded because they used VD or calcium carbonate and vitamin D_3_ tablets (Caltrate D). Finally, samples and clinical information for 88 individuals were analyzed. Based on the 25(OH)D concentrations in the serum of the subjects, the 88 participants were divided into an HVD group with 25(OH)D levels ≥ 20 ng/ml (n = 44) and an LVD group with 25(OH)D levels < 20 ng/ml (n = 44) (Fig. 1). The patients’ clinical characteristics, including age, weight, BMI, E2, OC, P1NP, CTX-1, PTH, Z score, T score and BMD of the LS, FN and total hip were comparable between the HVD and LVD groups. There were no differences in age, weight, BMI, E2, CTX-1, PTH, LS Z score, LS T score, LS BMD, FN Z score, FN T score, FN BMD, total hip Z score, total hip T score or total hip BMD between the two groups (Table 1 and Additional file 5: Table S1). Notably, there was a significant difference in serum 25(OH)D levels between the two groups (*p* < 0.001). The OC and P1NP levels were generally lower in the HVD group than in the LVD group (*p* < 0.05, respectively) (Table 1).

**Table 1:** Characteristics and clinical and biochemical index between HVD group (n=44) and LVD group (n=44)

Variables	HVD group (n=44)	LVD group (n=44)	P-value
Age (years)	59.28±4.93	57.5±4.68	0.097
BMI (kg/m^2^)	24.31±2.35	23.85±2.54	0.383
25(OH)VD (ng/ml)	27.10±5.02	14.42±3.28	<0.001
E2 (pmol/L)	34.22±22.53	32.92±18.36	0.750
OC (ng/ml)	20.74±9.97	26.4±11.36	0.002
CTX-1 (ng/ml)	0.42±0.25	0.52±0.28	0.104
P1NP (ng/ml)	51.68±26.21	72.76±33.76	0.001
PTH (pg/ml)	45.56±25.86	49.21±18.4	0.238
LS BMD(g/cm^3^)	0.95±0.18	0.99±0.18	0.238
FN BMD(g/cm^3^)	0.81±0.14	0.84±0.14	0.275
Total hip BMD(g/cm^3^)	0.86±0.14	0.88±0.15	0.428

The values represent mean ± S.D.

*BMI* body mass index, *LS* lumbar spine 1–4, *FN* femoral neck, *BMD* bone mineral density, *E2* estrogen, *25(OH)VD* serum 25-hydroxyvitamin D3, *OC* osteocalcin, *CTX-1* type I collagen cross-linked c-telopeptide, *P1NP* procollagen type 1 n-terminal propeptide, *PTH* parathyroid hormone

### Comparison of bacterial diversity between the HVD and LVD groups

**Fig. 2:**
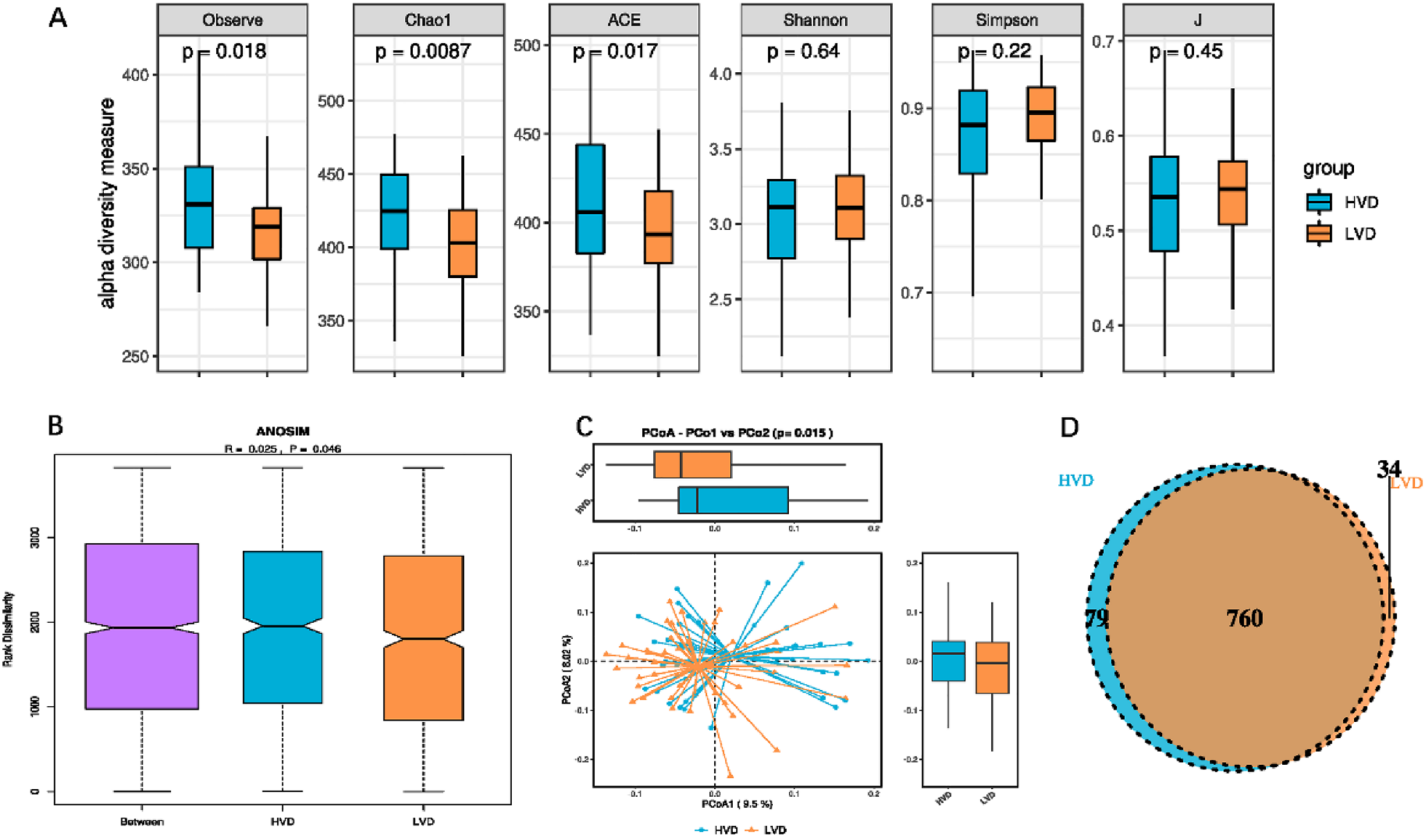
Decreased bacterial richness and diversity in the Low 25(OH)D group. **A** Comparison of bacterial alpha diversity indexes, including Observe, Chao1, ACE, Shannon, Simpson and J. **B** ANOSIM was used to analyze the significant differences in colony distribution between the High 25(OH)D group and the Low 25(OH)D group (R = 0.025, *p* = 0.046). **C** PCoA revealing the bacterial communities between the High 25(OH)D group and the Low 25(OH)D group on the PCoA1 vs. PCoA2 axis (*p* = 0.015). **D** Venn diagram showing the differences in bacterial community structures between samples from the High 25(OH)D group and the Low 25(OH)D group

The alpha diversity estimated by the Observe, Chao1 and ACE indexes was significantly lower in the LVD group than in the HVD group (*p* < 0.05, respectively). No statistically significant difference was observed between the HVD group and the LVD group in the J index, Simpson index or Shannon index (*p* > 0.05, respectively) (Fig. 2A). Analysis of similarities (ANOSIM) was used to compare the within- and between-group similarity through a distance measure to test the null hypothesis that the average rank similarity between samples within a group is the same as the average rank similarity between samples belonging to different groups. There were significant differences in colony distribution between the HVD group and the LVD group (Fig. 2B). PCoA was analyzed by using the distance matrix calculated from the species composition of the sample. The horizontal axis and the vertical axis represent the contribution rate of the first principal component (PCoA1) of 9.5% and the second principal component (PCoA2) of 8.02%, respectively (*p* = 0.015) (Fig. 2C). Venn diagram displaying the common and unique OTUs of the gut microbiota between the HVD and LVD groups. The size of the circle represents the number of OTUs. There were 760 common OTUs and 79 unique OTUs in the HVD group and 34 unique OTUs in the LVD group (Fig. 2D).

### Comparison of the bacterial community between the HVD and LVD groups

**Fig. 3:**
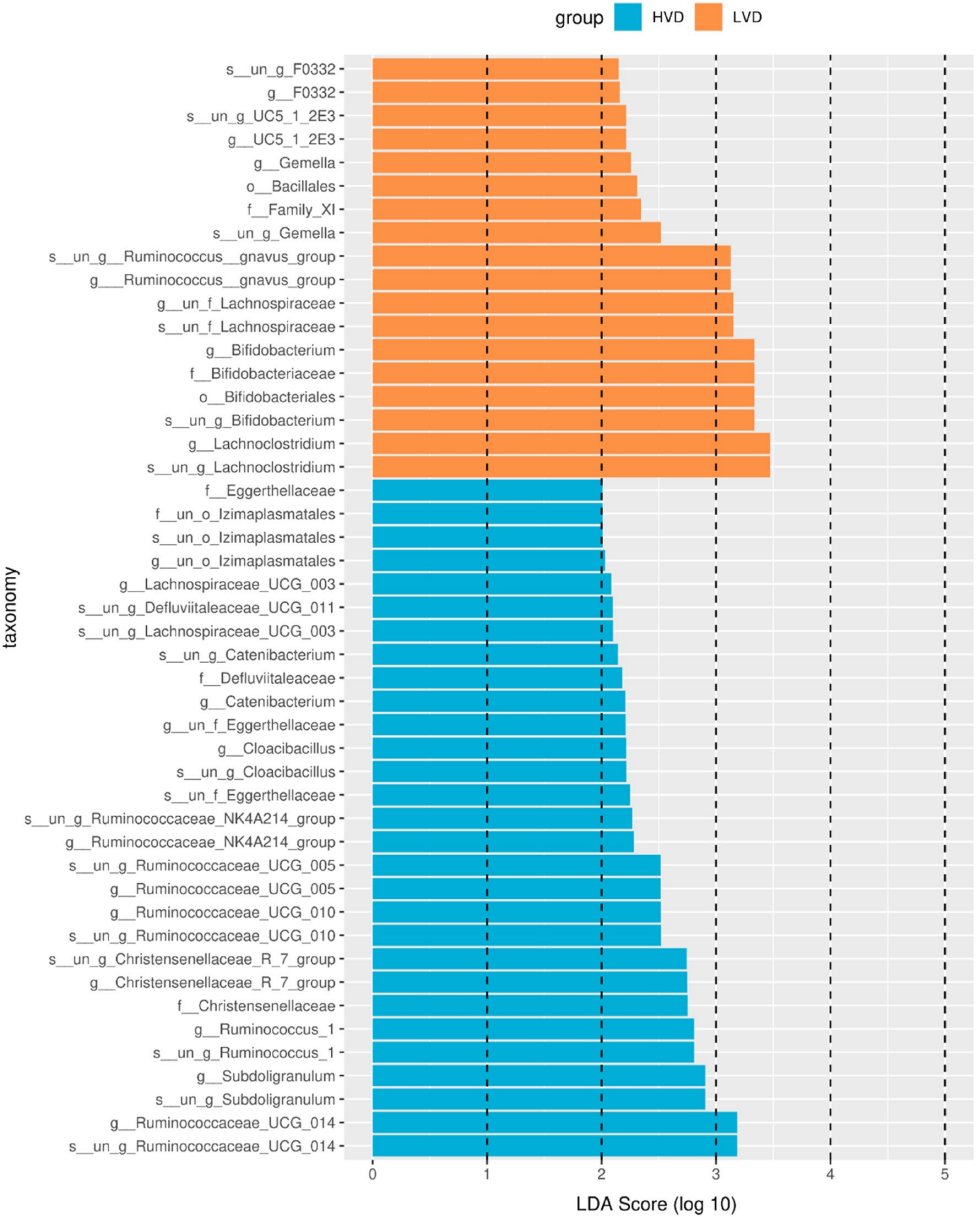
Discriminative taxa between the High 25(OH)D group and Low 25(OH)D group. LEfSe can be used to analyze the differences between groups and identify different microbial species, and this information can be used to develop biomarkers and promote other studies. In the above two syndrome types, the LDA threshold was set as 3 to screen the characteristic flora of the corresponding syndrome types

Linear discriminant analysis effect size can be used to analyze the differences between groups and identify different microbial species, which can be used to develop biomarkers and promote other studies. In the LVD group and the HVD group, the linear discriminant analysis (LDA) threshold was set as 3 to screen the characteristic flora of the corresponding groups. Two kinds of characteristic differences in microflora were analyzed in the HVD group, and 10 kinds of characteristic differences in microflora were analyzed in the LVD group (Fig. 3). We observed that *Christensenellaceae, Eggerthellaceae* and *Cloacibacillus* were enriched in the HVD group. G_*Bifidobacterium, g_F0332, g_Gemella, g_Lachnoclostridium, g_UC5*_*1*_*2E3, g_Ruminococcus_gnavus_group* and s_*un_f_Lachnospiraceae* were enriched in the LVD group (Fig. 3). The relative abundances of f_Family_XI, f_Bifidobacteriales, f_Bifidobacteriaceae, and o_Bacillales were significantly higher in the LVD group than in the HVD group. Furthermore, the relative abundances of f_Christensenellaceae, f_Eggerthellaceae, f_Defluviitaleaceae, and f_un_o_lzimaplasmatales were significantly higher in the HVD group than in the LVD group (Additional file 2: Fig. S2).

The results showed that the pathways associated with flavone and flavonol biosynthesis, pentose and glucoronate interconversions and cyanoamino acid metabolism were significantly enriched in the bacterial communities of the LVD group compared with those of the HVD group (Additional file 3: Fig. S3A). Furthermore, the 20 distinguished pathways were calculated to reveal the potential interaction mode (Additional file 3: Fig. S3B).

### Fecal metabolism profiles in the HVD and LVD groups

**Fig. 4:**
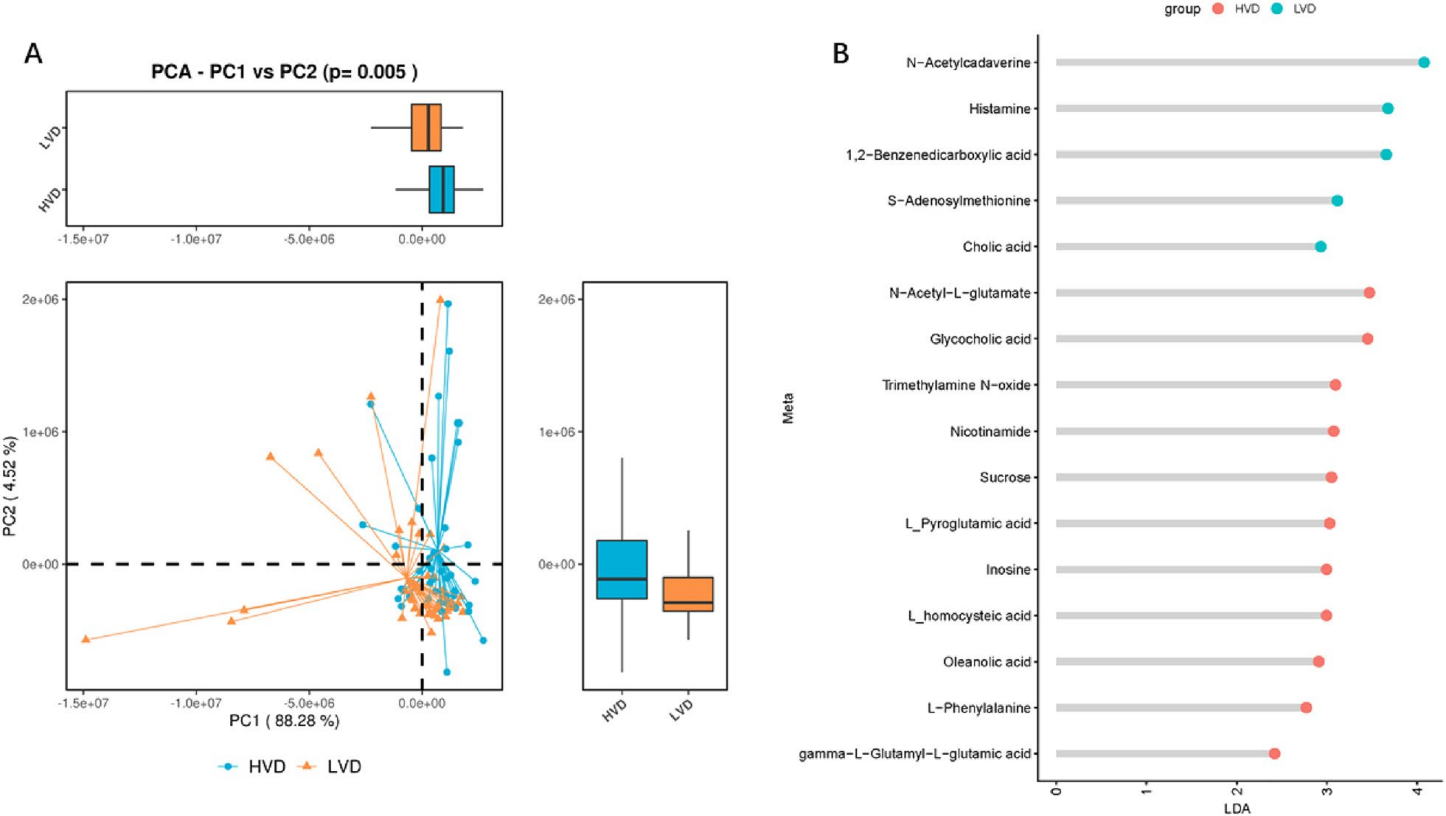
Discriminative fecal metabolites between the High 25(OH)D group and Low 25(OH)D group. **A** Principal Component Analysis (PCA) revealing the inter-individual variability of the fecal metabolites between the High 25(OH)D group and the Low 25(OH)D group on the PC1 vs PC2 axis (p = 0.005). **B** The x-axis shows the logarithms (base 10) of the linear discriminant analysis (LDA). The y-axis shows the discriminative fecal metabolites

To assess whether the profiles of fecal metabolites are associated with 25(OH)D levels, we performed metabolic sequencing of all stool samples. There were significant differences in fecal metabolites between the HVD group and the LVD group (Fig. 4A). Further stratified analysis by metabolite categories revealed that sucrose, l-pyroglutamic acid, inosine, l-homocysteic acid, oleanolic acid, N-acetyl-l-glutamate, glycocholic acid, trimethylamine N-oxide, nicotinamide, l-phenylalanine, and gamma-l-glutamyl-l-glutamic acid were significantly more abundant in the HVD group than in the LVD group. However, 1,2-benzenedicarboxylic acid, cholic acid, N-acetylcadaverine, histamine, and S-adenosylmethionine were significantly less abundant in the HVD group than in the LVD group (Fig. 4B).

### Association between featured bacterial taxa and metabolites

**Fig. 5:**
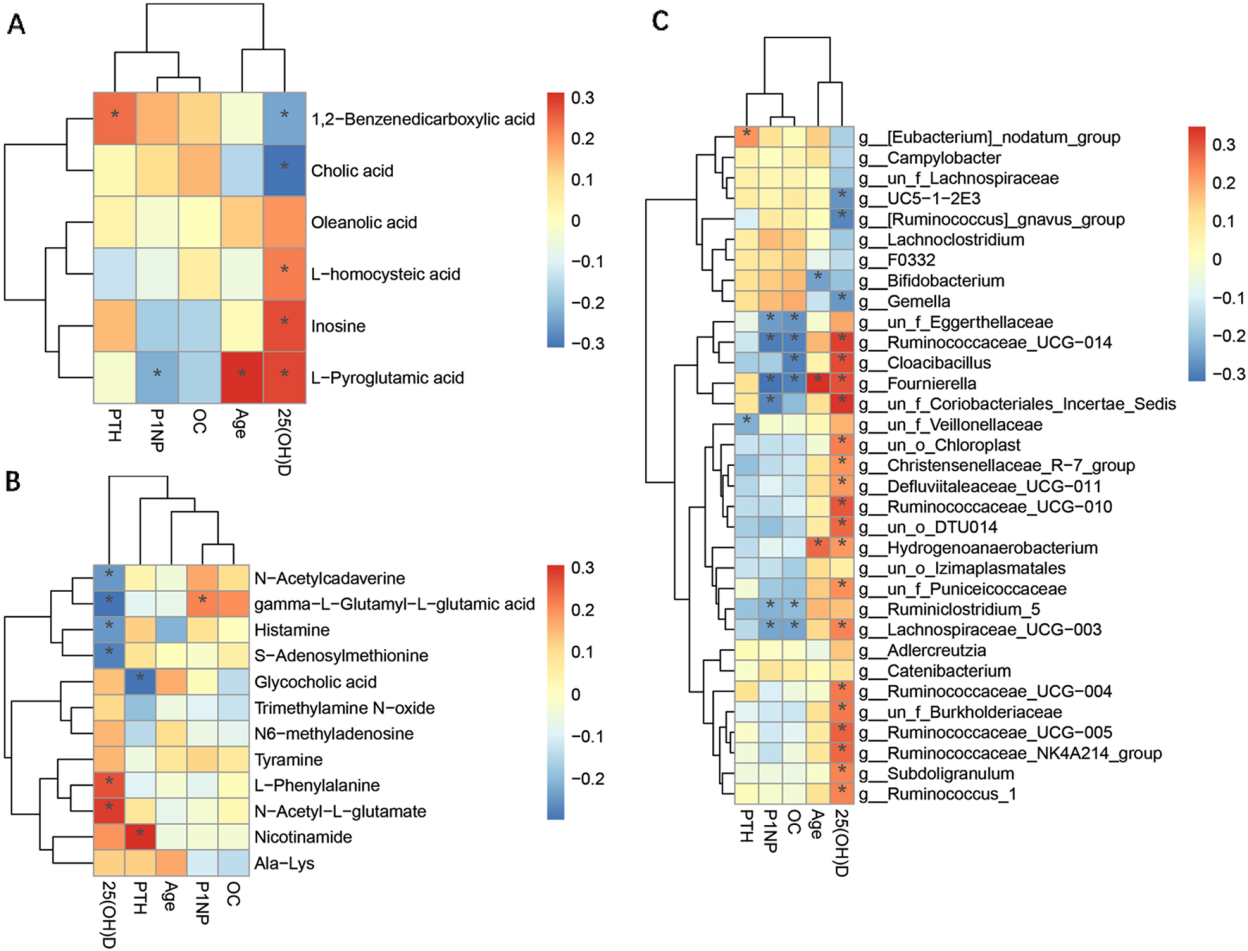
The correlation heatmap among the discriminative genera, discriminative fecal metabolites and the clinical indexes. **A**, **B** Heatmap for Spearman correlation analysis between fecal metabolites and clinical variables at the family level. **A** Negative modes. **B** Positive modes. X-axis, clinical variables. Y-axis, fecal metabolites. **C** Heatmap for Spearman correlation analysis between intestinal flora and clinical variables at the family level. X-axis, clinical variables. Y-axis, family. Correlation significance, *denotes *p* < 0.05

The relationships among the different bacteria, metabolites and clinical profiles were examined by correlation analysis (Spearman) to evaluate the relationship between the gut bacteria and clinical indexes and between the fecal metabolites and clinical indexes. We found that l-pyroglutamic acid, inosine, l-homocysteic acid, N-acetyl-l-glutamate, and l-phenylalanine correlated positively with 25(OH)D. 1,2-Benzenedicarboxylic acid, cholic acid, N-acetylcadaverine, gamma-l-glutamyl-l-glutamic acid, histamine, and S-adenosylmethionine were negatively correlated with 25(OH)D (Fig. 5A and B). There was a negative association between *g__UC5*_*1*_*2E3*, *g__[Ruminococcus]_gnavus_group*, *g__Gemella* and 25(OH)D. Conversely, there was a positive association between *g__Ruminococcaceae_UCG−014*, *g__Cloacibacillus*, *g__Fournierella*, *g__un_f_Coriobacteriales_Incertae_Sedis*, *g__un_o_Chloroplast, g__Christensenellaceae_R−7_group*, *g__Defluviitaleaceae_UCG−011*, *g__Ruminococcaceae_UCG−010*, *g__un_o_DTU014*, *g__Hydrogenoanaerobacterium*, g__un_f_Puniceicoccaceae, g__Lachnospiraceae_UCG−003, g__Ruminococcaceae_UCG−004, *g__un_f_Burkholderiaceae*, *g__Ruminococcaceae_UCG−005*, *g__Ruminococcaceae_NK4A214_group*, *g__Subdoligranulum*, *g__Ruminococcus_1* and 25(OH)D (Fig. 5C). Furthermore, the correlations between distinguished bacteria and different fecal metabolites are shown in Additional file 4: Fig. S4.

## Discussion

This study investigates associations among the gut microbiota, metabolites and serum 25(OH)D levels in postmenopausal women. Bacterial richness and diversity were decreased in postmenopausal women with low serum 25(OH)D (< 20 ng/ml). We also identified significant associations between different symbiotic gut microbial genera and serum 25(OH)D levels in this well-characterized cohort. Higher levels of sucrose, l-pyroglutamic acid, inosine, l-homocysteic acid, oleanolic acid, N-acetyl-l-glutamate, glycocholic acid, trimethylamine N-oxide, nicotinamide, l-phenylalanine, and gamma-l-glutamyl-l-glutamic acid were found in the HVD group. Furthermore, several distinguishing intestinal bacteria were associated with distinguishing metabolites related to serum 25(OH)D levels.

In a suburban area in China, over 60% of postmenopausal women had serum 25(OH)D levels over 20 ng/ml [36]. Low VD status is associated with greater bone turnover and bone loss. Women with hip fractures had significantly lower concentrations of serum 25(OH)D; higher concentrations of serum CTX-1, P1NP and OC; and lower FN and total hip BMDs [37]. Patients with nontraumatic osteonecrosis of the femoral head have been shown to have lower 25(OH)D levels and higher levels of P1NP, CTX-1 and OC [38]. In our study, postmenopausal females with lower levels of serum 25(OH)D also had higher OC, P1NP and CTX-1 levels. Due to estrogen deficiency, postmenopausal females have active bone turnover (bone resorption > bone formation) and increased bone loss [39].

Deep sequencing comparing pre- and postmenopausal women indicated that the gut microbiota displays different states before and after menopause. Compared to premenopausal women, postmenopausal women presented significantly lower richness and diversity [40]. Our results showed that postmenopausal women with high 25(OH)D levels had higher alpha diversity than the group with low 25(OH)D levels. A separate study showed inconsistent results: subjects with serum 25(OH)D concentrations below 50 nmol/L (n = 12) and subjects with serum 25(OH)D concentrations above 75 nmol/L (n = 10) had no significant differences in alpha diversity and beta diversity [41]. In contrast, another analysis of serum VD metabolites in 567 elderly men found that subjects with low 1,25(OH)_2_D levels had reduced bacterial richness and diversity [42]. These conflicting results may be related to the differences in the areas, sample sizes and populations.

There is evidence of a bidirectional interaction between VD and the gut microbiota in inflammation [17]. In rodents, VD regulates the composition and abundance of the gut microbiome, and 1,25(OH)_2_D_3_ or VDR deficiency leads to dysbiosis, increased inflammation, and higher susceptibility to intestinal injury [43]. In a meta-analysis of 8316 patients with inflammatory bowel disease (IBD), low levels of 25(OH)D were associated with increased odds of disease activity, mucosal inflammation, low quality of life, and clinical recurrence [44]. In this study, high serum 25(OH)D levels were positively correlated with *Subdoligranulum, Ruminococcaceae* and *Cloacibacillus*. At present, the direct relationship between these three bacterial groups and serum 25(OH)D levels in postmenopausal women is unknown. However, these three groups were found to comprise short-chain fatty acid (SCFA)-producing bacteria [45–47]. SCFAs play a key role in maintaining gut homeostasis and promoting organismal health by modulating dietary fiber and gut microbiota [48]. The abundance of *Subdoligranulum* has been shown to be significantly increased in patients with Crohn’s disease following one week of VD supplementation [49]. Research has shown that vitamin D_3_ positively correlates with hypertension-reduced bacterial genera, including *Subdoligranulum* and *Ruminiclostridium* [50].

Variations in the gut microbiota composition can interfere with VD status and biological functions [51]. Probiotic strains such as *Lactobacillus rhamnosus* and *Lactobacillus plantarum* increase VDR expression in both mouse and human intestinal epithelial cells [52]. In addition, probiotics *L. reuteri* and *Bifidobacterium longum* may increase BMD by promoting calcium absorption. Calcium absorption occurs throughout the intestine [53]. It has also been shown that the composition of the gut microbiota can influence the pH level of the gut [54], an important factor for nutrient absorption, especially calcium [55]. Moreover, some bacteria express enzymes involved in hydroxylation of steroids (i.e., Streptomyces griseolus with CYP105A1, Sebekia benihana with CYP-sb3a, Pseudonocardia autotrophica with Vdh) that are capable to hydroxylate and activate VD [56]. In humans, probiotics interfere with 25(OH)D levels and calcium intake and absorption and slightly decrease bone loss in elderly postmenopausal women to a magnitude quite similar to that observed with calcium and VD supplements [57]. In our study, *Bifidobacterium* and *Gemella* were negatively associated with low serum 25(OH)D levels. A study demonstrated that supplementation in 80 healthy VD-deficient women with a weekly oral dose of 50,000 IU vitamin D_3_ for 12 weeks significantly increased the diversity of the gut microbiota and the relative abundance of *Bifidobacterium* [17]. These findings indicate that several beneficial bacteria are associated with high serum 25(OH)D levels, but further studies are required to address questions on the potential detrimental impact and mechanisms of action in postmenopausal women. Moreover, VD supplementation may provide a benefit to the gut microbiota.

The findings of our study suggest that *Ruminococcus_1* and un_f_Puniceicoccaceae were positively correlated with both 25(OH)D and oleanolic acid levels. It has been reported that oleanolic acid is a triterpenoid with reported bone antiresorption activities. Oleanolic acid significantly increases BMD, improves microarchitectural properties, reduces urinary Ca excretion, and increases 1,25(OH)_2_D_3_ and renal CYP27B1 mRNA expression in mature ovariectomized mice [58]. Clinical biochemical indicators and bone density were also reversed by oleanolic acid treatment. A total of 25 potential biomarkers were identified in a rat model of glucocorticoid-induced osteoporosis, and oleanolic acid has a regulatory effect on 17 of them related to some vital metabolic pathways, such as linoleic acid metabolism, valine, leucine and isoleucine biosynthesis, phenylalanine, tyrosine and tryptophan biosynthesis and cysteine and methionine metabolism [59].

We found that cholic acid was negatively correlated with 25(OH)D levels. Various bile acids may play key roles in the control of calcium absorption. It has been shown that ursodeoxycholic acid promotes calcium absorption, whereas deoxycholic acid inhibits calcium absorption [60]. Indirectly, the gut microbiota can hinder vitamin activity through secondary bile acids, mainly lithocholic acid, which compete with VD for VDR binding and activation. In addition, lithocholic acid interacts with the VDR to induce CYP24A1 mRNA expression in the ileum, which leads to calcitriol inactivation [61]. l-Homocysteine has been observed to be positively correlated with 25(OH)D levels. A study from a large community-based cohort of asymptomatic adults found an inverse relationship between 25(OH)D and serum homocysteine levels among those with 25(OH)D concentrations of 21 ng/mL or less. However, no statistical decrease in serum homocysteine was observed once the 25(OH)D concentration rose above 21 ng/ml [62]. 25(OH)D and serum homocysteine have been shown to be significantly negatively correlated in postmenopausal nonosteoporotic females but not in osteoporotic females [63]. In vitro studies of murine preosteoblastic cells have indicated that 1,25-dihydroxyvitamin D_3_ is involved in the direct regulation of cystathionine beta synthase, an important enzyme in homocysteine metabolism [64].

Phenylalanine is an essential amino acid that is the raw material for the synthesis of tyrosine. Fecal and serum metabolomics analyses suggested that tyrosine and tryptophan metabolism and valine, leucine, and isoleucine degradation were significantly linked to the identified microbiota biomarkers and osteoporosis, respectively [65]. Osteopenia is observed in some patients affected by phenylalanine hydroxylase-deficient phenylketonuria [66]. Adult phenylketonuria patients had poor VD status and exhibited a predominance of bone resorption over bone formation [67]. High serum phenylalanine levels led to lower VD levels and bone loss, but we found that the levels of 25(OH)D and the fecal metabolite l-phenylalanine were positively correlated. These findings appear to be inconsistent, but much of the relevant existing literature is based on rodent studies, a small number of specimens or various diseases. However, the relationship among l-phenylalanine, l-homocysteine and VD metabolism in postmenopausal women is a valuable research direction.

Epidemiological evidence has indicated a relationship between VD and depression with anxiety [68, 69]. It has been reported that l-pyroglutamic acid possesses anxiolytic activity [70]. l-Pyroglutamic acid showed a positive correlation with 25(OH)D levels in our study. VD supplementation can improve anxiety symptoms in depressive patients with low VD levels after 6 months of intervention [71]. Vitamin D_3_ (5.0 mg/kg) administration seems to attenuate the anxiety-like profile in long-term ovariectomized adult rats subjected to chronic unpredictable mild stress [72]. It is suggested that VD could potentially be used as a therapeutic agent to prevent anxiety in postmenopausal women, and the mechanism of action of l-pyroglutamic acid requires further exploration.

There are several limitations to this study. First, taxonomic classifications of intestinal bacteria were analyzed based on 16S rRNA amplicon profiling rather than comprehensive shotgun metagenomic sequencing data. Second, it was a cross-sectional study. We only analyzed the correlation between 25(OH)D levels and gut microbiota, and identification of a causal relationship between multiple factors was not possible. Third, all included subjects were recruited from Xiamen, a central city along the southeastern coast of China. There is a single source of subjects, and the topography, climatic characteristics, and dietary patterns are similar. The results of this analysis may only reflect the situation of postmenopausal women in this region, which is not representative. In the future, a multicenter study incorporating a larger sample size could be conducted to validate this result. In addition, more in-depth exploration studies are necessary to understand the underlying mechanism of the relationship between the gut microbiome and VD metabolism in postmenopausal women.

## Conclusion

In summary, we identified the disordered profiles of the gut microbiota and fecal metabolomes in postmenopausal women with high or low serum 25(OH)D levels. The results were consistent in identifying associations between 25(OH)D levels and distinguishing bacteria and metabolites, and the relationships among them and the bone metabolic index were discussed. These differences provide strong evidence that specific genera within the gut represent a link between intestinal bacteria and serum 25(OH)D levels and could subsequently affect the health of postmenopausal women.

## Supplementary Information

**Additional file 1: Figure S1.** Rarefaction curves in observed_species of all the samples. The rarefaction curves constructed from the sequenced data has been basically stable, indicating that the sequenced data has benn basically stable at this sequencing depth.

**Additional file 2: Figure S2.** The taxonomic representation of statistically and biologically differences between High 25(OH)D group and Low 25(OH)D group. The color of discriminative taxa represents the taxa is more abundant in the corresponding group (High 25(OH)D group in blue, Low 25(OH)D group in orange).

**Additional file 3: Figure S3.** Functional PICRUSt analysis among the LVD and HVD group. Kyoto Encyclopedia of Genes(A) and Genomes and cluster of orthologous group(B) was used to further investigate the mechanism of intestinal flora.

**Additional file 4: Figure S4.** Heat map for spearman correlation analysis between fecal metabolites and discriminative genera at the family level. Only significant values (p < 0.05 after FDR adjustment) are shown. Orange and blue colors represent significant positive correlations and negative correlations. Darker color represents stronger correlations.

**Additional file 5: Table S1.** Characteristics and clinical and biochemical indexes between HVD group (n=44) and LVD group (n=44).

## Data Availability

The datasets generated during and analyzed during the current study are available from the corresponding author on reasonable request. All sequence data is available from NCBIs Sequence Read Archive (SRA), BioProject ID: PRJNA814014.
