# Supplementary material for: Association of serum 25-hydroxyvitamin D (25(OH)D) levels with the gut microbiota and metabolites in postmenopausal women in China
Source: Microb Cell Fact. 2022 Jul 11;21:137. doi: 10.1186/s12934-022-01858-6 (PMC9275287; doi:10.1186/s12934-022-01858-6)
Supplement: Supplementary file 1 — Additional file 1: Figure S1. Rarefaction curves in observed_species of all the samples. The rarefaction curves constructed from the sequenced data has been basically stable, indicating that the sequenced data has benn basically stable at this sequencing depth. [file 12934_2022_1858_MOESM1_ESM.docx]

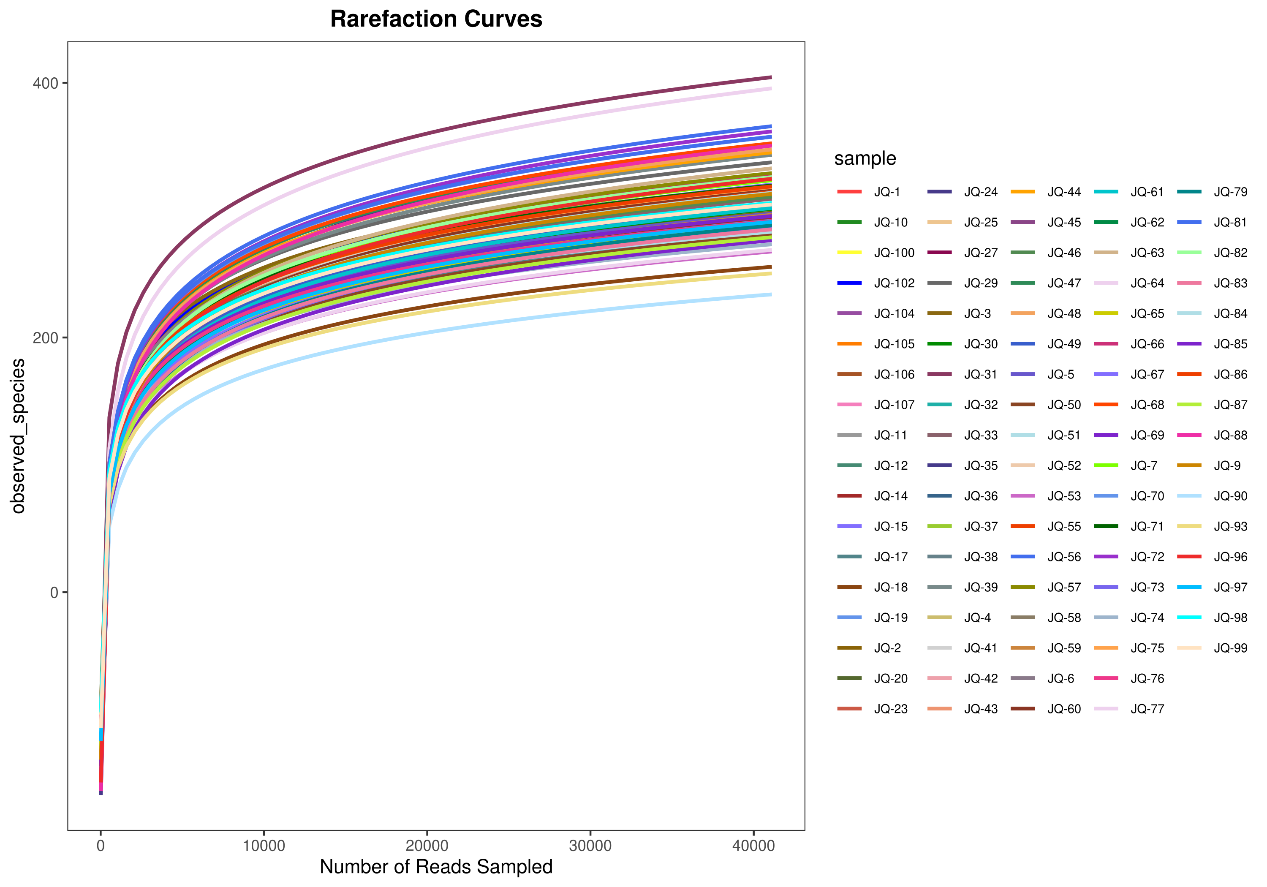


**Fig. S1 Rarefaction curves in observed_species of all the samples.** The rarefaction curves constructed from the sequenced data has been basically stable, indicating that the sequenced data has been basically stable at this sequencing depth.
