# Supplementary material for: Association of serum 25-hydroxyvitamin D (25(OH)D) levels with the gut microbiota and metabolites in postmenopausal women in China
Source: Microb Cell Fact. 2022 Jul 11;21:137. doi: 10.1186/s12934-022-01858-6 (PMC9275287; doi:10.1186/s12934-022-01858-6)
Supplement: Supplementary file 2 — Additional file 2: Figure S2. The taxonomic representation of statistically and biologically differences between High 25(OH)D group and Low 25(OH)D group. The color of discriminative taxa represents the taxa is more abundant in the corresponding group (High 25(OH)D group in blue, Low 25(OH)D group in orange). [file 12934_2022_1858_MOESM2_ESM.docx]

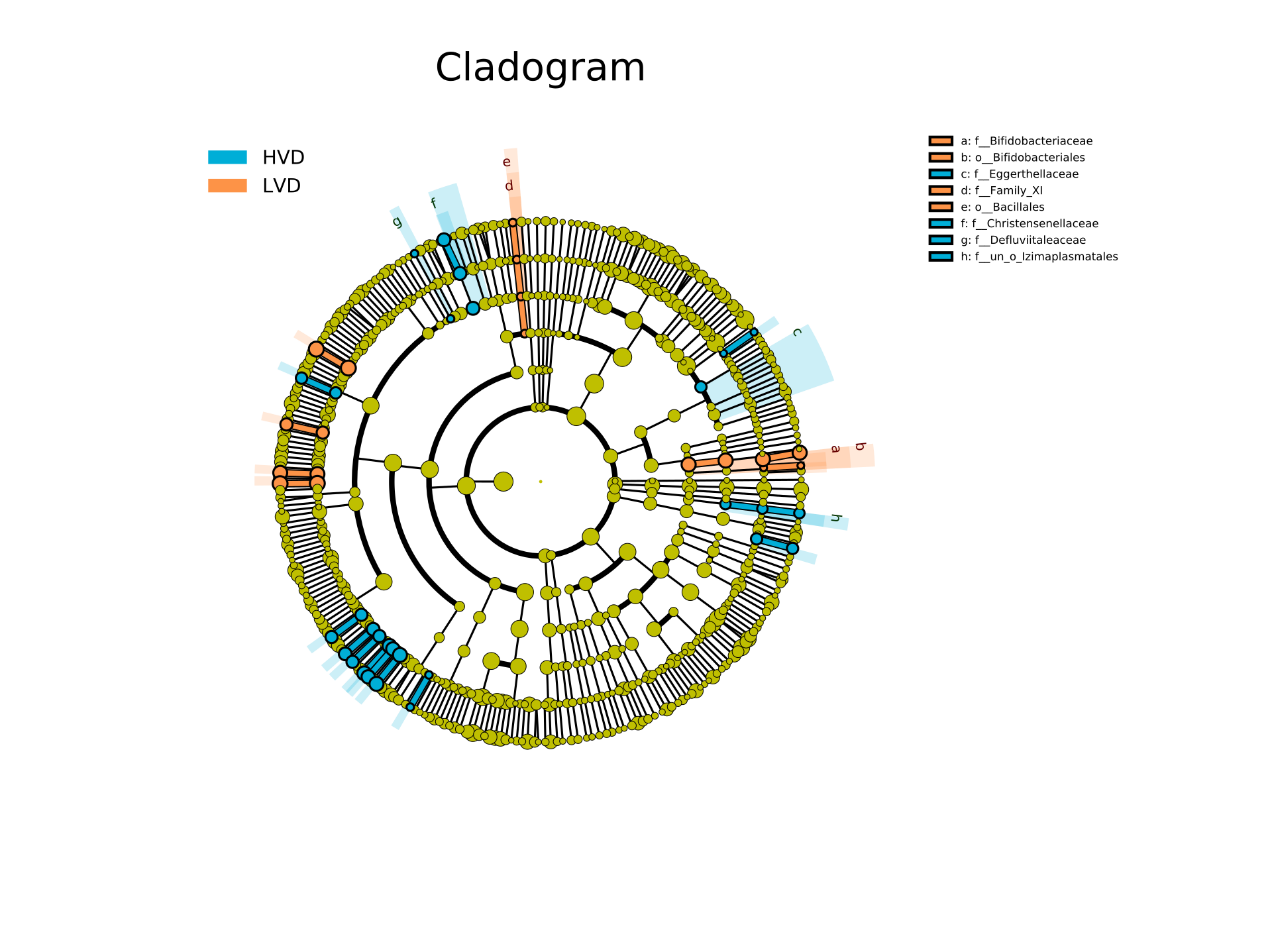


**Fig. S2 The taxonomic representation of statistically and biologically differences between High 25(OH)D group and** **Low 25(OH)D group.** The color of discriminative taxa represents the taxa is more abundant in the corresponding group (High 25(OH)D group in blue, Low 25(OH)D group in orange).
