# Supplementary material for: Association of serum 25-hydroxyvitamin D (25(OH)D) levels with the gut microbiota and metabolites in postmenopausal women in China
Source: Microb Cell Fact. 2022 Jul 11;21:137. doi: 10.1186/s12934-022-01858-6 (PMC9275287; doi:10.1186/s12934-022-01858-6)
Supplement: Supplementary file 3 — Additional file 3: Figure S3. Functional PICRUSt analysis among the LVD and HVD group. Kyoto Encyclopedia of Genes(A) and Genomes and cluster of orthologous group(B) was used to further investigate the mechanism of intestinal flora. [file 12934_2022_1858_MOESM3_ESM.docx]

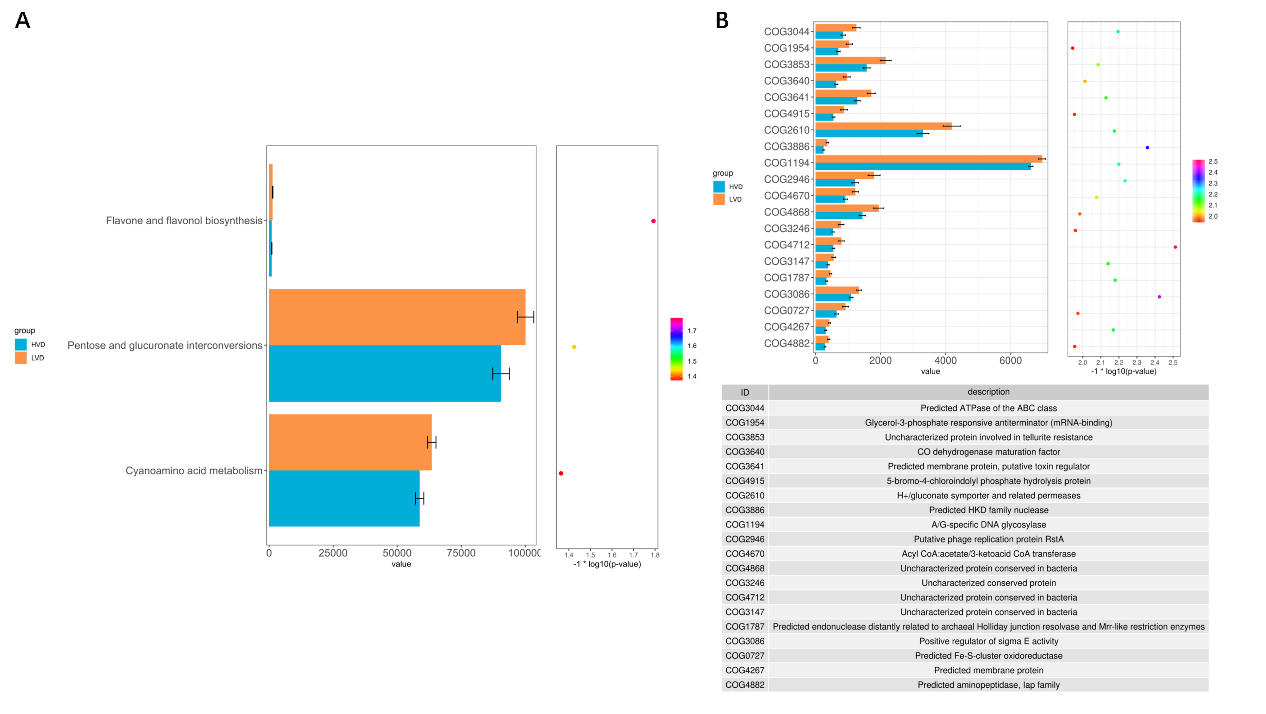


**Fig. S3 Functional PICRUSt analysis among the LVD and HVD group.** Kyoto Encyclopedia of Genes (A) and Genomes and cluster of orthologous group (B) was used to further investigate the mechanism of intestinal flora.
