# Supplementary material for: Association of serum 25-hydroxyvitamin D (25(OH)D) levels with the gut microbiota and metabolites in postmenopausal women in China
Source: Microb Cell Fact. 2022 Jul 11;21:137. doi: 10.1186/s12934-022-01858-6 (PMC9275287; doi:10.1186/s12934-022-01858-6)
Supplement: Supplementary file 4 — Additional file 4: Figure S4. Heat map for spearman correlation analysis between fecal metabolites and discriminative genera at the family level. Only significant values (p < 0.05 after FDR adjustment) are shown. Orange and blue colors represent significant positive correlations and negative correlations. Darker color represents stronger correlations. [file 12934_2022_1858_MOESM4_ESM.docx]

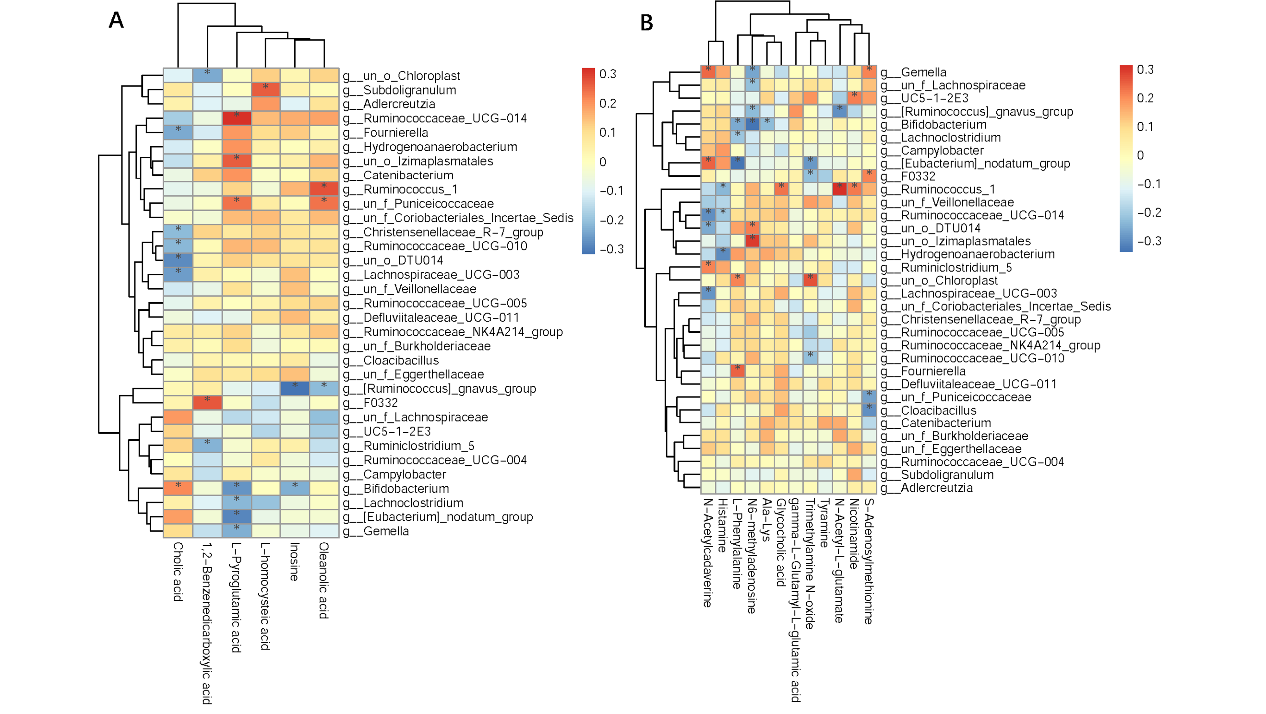


**Fig. S4** **Heat map for spearman correlation analysis between fecal metabolites and discriminative genera at the family level.** Only significant values (p < 0.05 after FDR adjustment) are shown. Orange and blue colors represent significant positive correlations and negative correlations. Darker color represents stronger correlations.
